# Creatine Supplementation Practices in Saudi Arabia: Prevalence, Patterns, and Health Implications

**DOI:** 10.7759/cureus.94947

**Published:** 2025-10-19

**Authors:** Mohammad Othman, Abdullah Aljehani, Saad Alzahrani, Muhana Alzahrani, Feras Monusar, Mosab Alshammari

**Affiliations:** 1 Clinical Sciences, Fakeeh College for Medical Sciences, Jeddah, SAU; 2 Medicine, Fakeeh College for Medical Sciences, Jeddah, SAU

**Keywords:** creatine, dietary supplements, ergogenic aids, nutrition education, prevalence, saudi athletes

## Abstract

Background

Creatine monohydrate is a well-known ergogenic aid that increases phosphocreatine resynthesis and improves performance during high-intensity exercise. Although common, the creatine supplementation practices of young Saudi athletes are unknown.

Objective

The objective of this study was to assess the prevalence, use patterns, and sources of information of creatine supplementation and to investigate demographic and behavioral determinants of supplement use among Saudi athletes aged 18-25 years.

Methods

A cross-sectional survey questionnaire of 326 athletes from Jeddah provided data on sport supplement types taken, dosing regimen, self-reported side effects, main advisers, and nutrition attitudes. Using IBM SPSS Statistics software, version 28 (IBM Corp., Armonk, NY), chi-square tests were employed to test associations of supplement intake with gender, frequency of exercise, importance attached to nutrition, and participation at workshops.

Results

Among the respondents, 30.4% reported any sport supplement use, 5.8% of whom took creatine. Amongst users of supplements, 30.7% did not have any knowledge of dosage. Coaches (31.9%) and nutritionists (22.4%) were the chief advisers, while 15.6% of participants attended a formal workshop. Supplementation use was significantly associated with male gender (χ²(1)=11.899, p<0.001), exercise (χ²(1)=37.159, p<0.001), high nutrition value placing (χ²(2)=21.341, p<0.001), and attendance at workshops (χ²(1)=6.665, p=0.010).

Conclusion

Creatine supplementation was less than internationally reported in young Saudi Arabian athletes. Confusion and misuse of high dosing, as well as reliance on non-expert recommendations, highlight the need for education by dietitians and more effective regulatory advice on supplement labeling to promote safe, evidence-based usage.

## Introduction

Creatine is an endogenously produced guanidine compound whose exogenous sources are meat and fish [1]. Creatine and its phosphate analogue phosphocreatine serve as the skeletal muscle's immediate high-energy phosphate donors to resupply adenosine triphosphate (ATP) in high-intensity, short-term exercise [2]. Creatine monohydrate supplementation, typically used via a loading regimen of 0.3 g/kg/day for five to seven days and 0.03 g/kg/day maintenance, has also been shown to augment intramuscular creatine and phosphocreatine stores, respectively, enhancing peak power output, strength gains, and training volume [3, 4].

In addition to its ergogenic effect, recent research suggests that creatine supplements may also be of value in the recovery from exercise, brain performance, and certain clinical populations without notable side effects for healthy athletes when supplemented according to best practices [5, 6]. Being one of the most scientifically studied and top-selling sport supplements, creatine has the backing of professional societies such as the International Society of Sports Nutrition, which attests to its safety and efficacy for both therapeutic and athletic applications [6].

In Saudi Arabian gym members and athletes, dietary supplement use has ranged between 33.9% and 37.8% [7-9]. The most common ergogenic supplement after whey protein and amino acids is creatine [8]. Reasons for use are to improve body composition, overall well-being, and performance [7, 10]. Relying on lay sources (peers, coaches, and online sources) is both dosing validity and side-effect problematic, particularly in the context of advice to avoid creatine supplementation in people under 18 years of age [11].

Despite its worldwide availability and acceptability, there is limited systematic information on the prevalence, pattern of use, and health effects of creatine supplementation in young Saudi athletes. To address this gap, the present study aims to do the following: Primary aim: to determine the prevalence and dosing patterns of creatine supplement use among athletes aged 18-25 years in Jeddah. Secondary aim: to identify demographic and behavioral factors associated with creatine use. Findings will inform selective educational interventions, policy development, and clinical practice to optimize safe and evidence-based creatine supplementation among the Saudi sporting community.

## Materials and methods

Study design

This cross-sectional study was conducted between January and June 2025 using an anonymous, self-completed internet-based survey distributed via leisure and sporting centers to athletes using a convenience sampling method (Appendix A).

Participants

Since most of the previous studies conducted in many cities in Saudi Arabia, except Jeddah, have reported the prevalence ranging between 65.6% and 36.1% for different sports supplements, based on the average of previous similar studies (for sports supplements), this research estimated a 50% prevalence, 95% confidence, and 5% margin of error. Considering a 20% nonresponse or incomplete form rate, this research aimed to recruit 310 participants. Three hundred and twenty-six participants were recruited in leisure and sporting centers. Participants were 18-25-year-olds participating in competitive sporting activities attending a sports center. This research planned to report on any refusals and missing data of participants' surveys, which were not seen in this study.

Instrument

The survey instrument was adapted from Aljaloud & Ibrahim (2013), a pre-existing validated instrument (which is publicly available and free to adapt with citation) [7]. The instrument contained items on demography, exercise profile, supplement use, information and knowledge sources, and nutrition attitudes. Questionnaires were prepared on Google Forms (Google LLC, Mountain View, CA), and incomplete forms and invalid forms were identified, reported, and excluded from the analysis. 

Analysis

Participants provided electronic informed consent before responding to the survey. All the information was collected anonymously and stored on a secure, password-protected computer. Analyses were performed using IBM SPSS Statistics software, version 28 (IBM Corp., Armonk, NY). Descriptive statistics (frequencies, percentages) provided participant descriptions and supplemented habits. Association correlations between supplement use and categorical predictors were evaluated using chi-square tests; continuous variables (e.g., age) were compared with independent t-tests. P ≤ 0.05 was regarded as statistically significant.

Ethics

The Fakeeh College for Medical Sciences Ethics Committee, Jeddah, Saudi Arabia, cleared this study (approval number: FIRB-25-0122). Electronic informed consent was obtained from all participants, and confidentiality was ensured through the anonymized collection and secure storage of the data.

## Results

The sample size was 326. Sociodemographic characteristics of the study sample are presented in Table 1. Most of the participants were men (53.9%), while 46.0% were females. Most participants were between 26 and 35 years old (29.1%), followed by participants between 19 and 25 years old (25.5%) and 36 and 45 years old (22.4%). Most respondents were Saudi nationals (90.2%). According to BMI categories, 39.9% were of normal weight, 28.2% overweight, 26.1% obese, and 5.8% underweight. More than half (55.8%) reported exercising regularly.

**Table 1:** Sociodemographic Characteristics of the Participants (N = 326)

Variable	Category	Frequency (n)	Percentage (%)
Gender	Male	176	53.9
	Female	150	46.0
Age Group (years)	18	25	7.7
	19–25	83	25.5
	26–35	95	29.1
	36–45	73	22.4
	Over 45	50	15.3
Nationality	Saudi	294	90.2
	Non-Saudi	32	9.8
BMI Category	Underweight	19	5.8
	Normal Weight	130	39.9
	Overweight	92	28.2
	Obese	85	26.1
Do You Exercise Regularly?	Yes	182	55.8
	No	144	44.2

Participant views regarding nutrition and supplementation are summarized in Table 2. Nearly half (47.8%) reported "very important" as their score for good nutrition, and 30.4% consumed sports supplements. The most common reasons given for taking supplements were to enhance health (38.7%), physical appearance (19.0%), and performance (18.4%). Supplements were predominantly purchased at pharmacies (40.8%), followed by sports stores or gyms (34.7%) and online stores (21.8%). A small proportion (2.8%) reported not purchasing or using supplements. The main sources of information regarding supplements were coaches (31.9%) and nutritionists (22.4%). Only 15.6% of participants attended a supplement workshop, with 60.4% having access to a dietitian.

**Table 2:** Nutrition and Supplement Use Among Participants (N = 326)

Variable	Category	Frequency (n)	Percentage (%)
Importance of Good Nutrition	Very Important	156	47.8
	Important	128	39.3
	Not Important	42	12.9
Uses Sports Supplements	Yes	99	30.4
	No	227	69.6
Main Reason for Supplement Use	Improve Health	126	38.7
	Enhance Performance	60	18.4
	Physical Appearance	62	19.0
	Recovery	30	9.2
	Injury Prevention	48	14.7
Source of Supplement Purchase	Online Store	71	21.8
	Sports Store/Gym	113	34.7
	Pharmacy	133	40.8
	Does Not Use/Buy	9	2.8
Source of Supplement Information	Online	1	0.3
	Coach	104	31.9
	Nutritionist	73	22.4
	Doctor	23	7.1
	Friends/Family	2	0.6
	Media	4	1.2
	None	119	36.5
Attended Supplement Workshop	Yes	51	15.6
	No	275	84.4
Access to Dietitian	Yes	179	60.4
	No	129	39.6

Supplement use patterns are listed in Table 3. Most participants (52.1%) used supplements not otherwise specifically stated (labeled as "other"), followed by vitamins and minerals (15.3%) and protein supplements (9.8%). Supplement use was most likely before exercise (57.0%). The most frequently reported reasons for supplement use were to increase energy (37.1%) and to treat medical or nutritional deficiencies (14.4%). In terms of dosage, 32.0% reported taking a moderate dose (three to five units), and 30.7% were not sure of the dosage.

**Table 3:** Supplement Use Patterns Among Participants (N = 326)

Variable	Category	Frequency (n)	Percentage (%)
Type of Supplement Used	Protein	32	9.8
	Creatine	19	5.8
	Amino Acids	1	0.3
	Omega-3/Fish Oil	2	0.6
	Vitamins/Minerals	50	15.3
	Other	170	52.1
	No Supplement	52	16.0
Timing of Supplement Intake	Before Workout	186	57.0
	During Workout	41	12.6
	After Workout	99	30.4
Reason for Supplement Use	Energy	121	37.1
	Medical/Deficiency	47	14.4
	Recovery	24	7.4
	Immune Support	17	5.2
	Peer Influence	7	2.1
	None	110	33.7
Supplement Dosage Category	Low (1 unit)	51	15.6
	Moderate (2 units)	66	20.2
	Medium (3–5 units)	104	32.0
	High (>5 units)	5	1.5
	I Don’t Know	100	30.7

Chi-square tests of independence revealed several statistically significant associations (Table 4). The use of supplements was strongly associated with gender (χ²(1) = 11.899, p < .001), regular exercise (χ²(1) = 37.159, p < .001), and viewing nutrition as very important (χ²(2) = 21.341, p < .001). Participants who had ever been to a supplement workshop were more likely to use supplements (χ²(1) = 6.665, p = .010). In addition, nationality was significantly correlated with exercising regularly (χ²(1) = 4.106, p = .043). Significant associations between demographic factors and supplement use behaviors, identified through chi-square analysis (p < 0.05).

**Table 4:** Summary of Significant Chi-square Associations (N = 326)

Variable 1	Variable 2	χ²	df	p-value
Gender	Supplement Use	11.899	1	< .001
Exercise	Supplement Use	37.159	1	< .001
Importance of Nutrition	Supplement Use	21.341	2	< .001
Attended Workshop	Supplement Use	6.665	1	0.01
Nationality	Exercise	4.106	1	0.043

## Discussion

Use of supplements

The present study showed that approximately 30% of young Saudi athletes (aged 18-25 years) took sport supplements, which is much lower than the 48.9%-87% range reported around the world over the past decade [12-15]. Regionally, Saudi studies have described use rates of between 33.9% and 93.3% [7, 16]. This research's comparatively lower prevalence might be explained by sampling differences (school-based vs. gym-based populations), supplement availability, or evolving health attitudes within the target group.

Gender and behavioral correlations

Consistent with international surveys, male athletes in this study sample were more likely than female athletes to use supplements and to favor performance-enhancing categories such as protein and creatine [17, 18]. Female respondents, while smaller in number, were tending towards multivitamins and minerals, aligning with trends in the literature [19]. The strong relationship between high exercise frequency, positive appreciation of nutrition, and supplement use aligns with work showing that athletes with greater sports engagement and positive efficacy beliefs are more inclined to supplement [20, 21].

Supplement types, dosage, and information sources

In this cohort, the leading "other" category suggests that locally popular formulations (e.g., herbal blends) may play a stronger role than in Western samples, where multivitamins/minerals, protein, and creatine are the most commonly used supplements in adolescents [22, 23]. On the other hand, the 30.7% dosing uncertainty that was observed is consistent with reports that most young athletes are given no clear dosing instructions for safety and efficacy [24, 25]. The primary sources for information and advice were coaches (31.9%) and nutritionists (22.4%). This finding suggests traditional reliance on face‐to‐face mentors [12, 13], amid global trends of growing internet and social media influence among teenagers [14, 26].

Education and workshop effect

Only 15.6% of this research sample had attended a formal supplement workshop, yet workshop attendance was closely correlated with supplement use. Therefore, previous findings suggest that formal education both raises awareness and may unintentionally legitimize supplementation. Previous interventions demonstrate that dietitian‐led programs can increase knowledge and safe practices [27, 28], yet underutilization of these services persists. The inclusion of certified sports nutrition professionals within sporting contexts could both support informed decision-making and ameliorate misuse.

Cultural and regulatory context

Performance enhancement, health maintenance, and body ideals are prominent cultural drivers among Saudi cohorts [16, 29]. Education level, exercise frequency, and household income are positively associated with greater use, patterns duplicated in these results [10]. Regulatory oversight of supplement labeling and safety for adolescents remains weak [30, 31]. This suggests increased quality testing, more specific age‐appropriate recommendations, and stronger labeling standards to protect young athletes [31, 32].

Health risks and ethical considerations

This research finding of widespread dosage uncertainty underscores the ethical imperative for balanced‐diet promotion and risk‐message strategies targeted to adolescents. While some supplements offer benefits such as faster recovery or nutrient support [33]. Teenagers face unique risks, including exceeding safe-level nutrient levels, medication interactions, and gateway exposures to illicit drugs [12, 34].

Limitations

The cross-sectional design has limited reach. In addition, reliance on self-reported data increases the possibility of recall bias and social desirability risk. The convenience sample of athletes, made up of gym-based athletes, prevents generalizability to school-based or recreational populations. The lack of supplement duration data and product confirmation prevents the assessment of cumulative exposure and potential adulteration. The analytical plan, limited to bivariate chi-square tests, ignores confounding between gender, exercise frequency, and nutrition attitudes, which are interdependent behaviors that limit the establishment of determinants of supplement use. Finally, with no explanation of workshop content, it is not possible to sufficiently determine the effectiveness of educational interventions.

Recommendations

Prospective longitudinal studies are needed to untangle temporal relationships between supplement use, health effects, and progression to performance-enhancing drugs. Randomized trials of dietitian-led, coach-supported workshops must identify curricula that optimize dosing correctness and risk awareness. Future surveys must employ more diverse sampling frames, e.g., school teams and community clubs, and incorporate objective product verification in collaboration with regulatory agencies. National standards on age labeling, standardized dosage instructions, and advertising restrictions must be established and implemented to safeguard young athletes.

## Conclusions

This study is one of the first to document creatine supplement use among Saudi adult athletes. While overall supplement use and creatine use are lower than reported in most international studies, there are important safety and education gaps. Uncertainty on high doses, consulting informal advisers (peers and coaches), and low consultation with formal workshops reveal the need for directed interventions. Gender, regular training, intense consideration of nutrition, and attendance at workshops were identified as strong predictors of supplement use, i.e., education and behavior are equally contributing factors in making decisions.

To promote safe, evidence-based creatine use, physicians and sports medicine clinics must integrate certified registered dietitians into training programs, create age-sensitive nutrition education curricula, and work with governing agencies to standardize labeling and dosing instructions. Longitudinal designs should be used in future research to monitor health status over time and to measure the impact of formal education on supplement knowledge and behavior. Fulfillment of these agendas will optimize performance gains while safeguarding long-term youth athlete health.

## Figures and Tables

**Figure 1:**
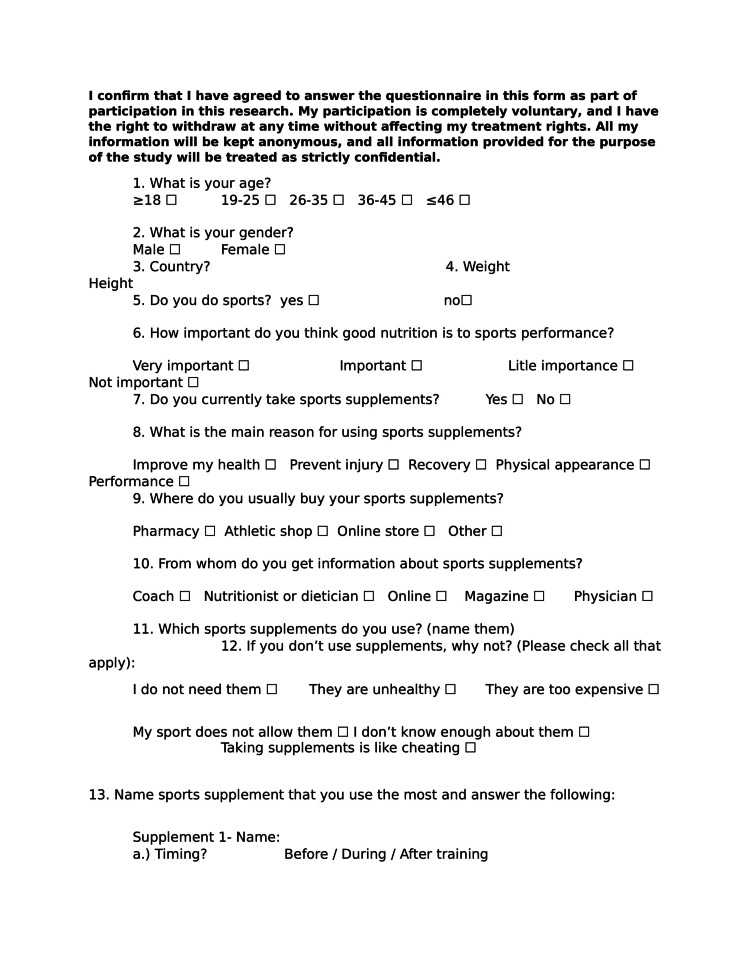
Research Questionnaire Part 1

**Figure 2:**
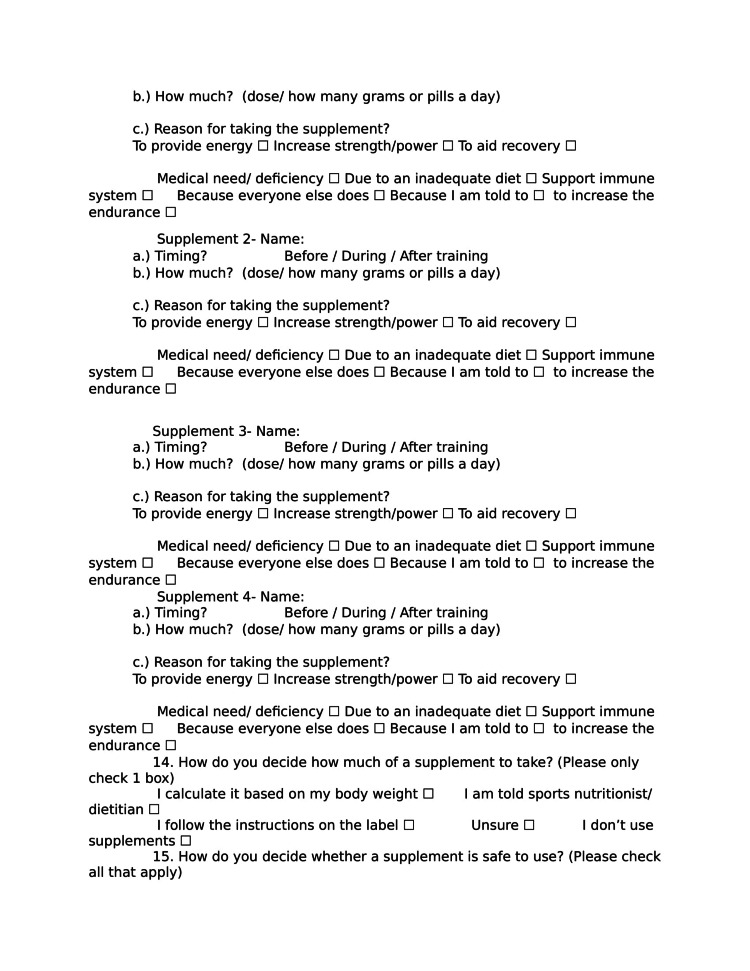
Research Questionnaire Part 2

**Figure 3:**
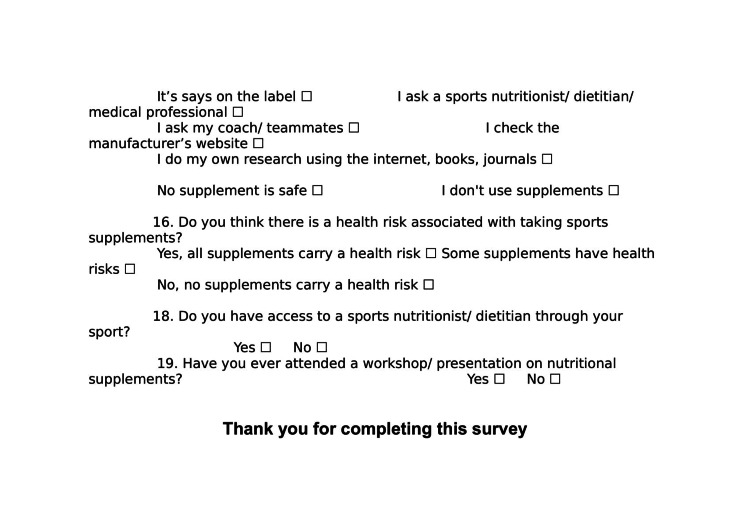
Research Questionnaire Part 3

## Appendices

Appendix A

## References

[1] Júnior MP, Moraes A de JP de, Ornellas FH (2012). Efficiency of creatine supplementation on human physical performance (Article in Portuguese). Braz J Exerc Prescr Physiol.

[2] Cooper R, Naclerio F, Allgrove J, Jimenez A (2012). Creatine supplementation with specific view to exercise/sports performance: an update. J Int Soc Sports Nutr.

[3] Hall M, Trojian TH (2013). Creatine supplementation. Curr Sports Med Rep.

[4] Kreider RB (2003). Effects of creatine supplementation on performance and training adaptations. Mol Cell Biochem.

[5] Mimansa D, Dahal K, Pokhrel NR, Kutal D (2024). Creatine supplements: what the research says about how it can help healthy athletes. Glob J Health Sci.

[6] Kreider RB, Kalman DS, Antonio J (2017). International Society of Sports Nutrition position stand: safety and efficacy of creatine supplementation in exercise, sport, and medicine. J Int Soc Sports Nutr.

[7] Aljaloud SO, Ibrahim SA (2013). Use of dietary supplements among professional athletes in Saudi Arabia. J Nutr Metab.

[8] Jawadi AH, Addar AM, Alazzam AS (2017). Prevalence of dietary supplements use among gymnasium users. J Nutr Metab.

[9] Aljaloud S, Al-Ghaiheb A, Wajid S (2020). Effect of athletes’ attitudes, beliefs, and knowledge on doping and dietary supplementation in Saudi sports clubs. J Musculoskelet Surg Res.

[10] Alfawaz H, Khan N, Alfaifi A (2017). Prevalence of dietary supplement use and associated factors among female college students in Saudi Arabia. BMC Womens Health.

[11] Metzl JD, Small E, Levine SR, Gershel JC (2001). Creatine use among young athletes. Pediatrics.

[12] Jovanov P, Đorđić V, Obradović B, Barak O, Pezo L, Marić A, Sakač M (2019). Prevalence, knowledge and attitudes towards using sports supplements among young athletes. J Int Soc Sports Nutr.

[13] Tawfik S, El Koofy N, Moawad EM (2016). Patterns of nutrition and dietary supplements use in young Egyptian athletes: a community-based cross-sectional survey. PLoS One.

[14] Waller MC, Kerr DA, Binnie MJ (2019). Supplement use and behaviors of athletes affiliated with an Australian state-based sports institute. Int J Sport Nutr Exerc Metab.

[15] Mas MF, Rañal JL, Rosario Concepción RA, González-Sepúlveda L, Rivas-Tumanyan S, Frontera WR, Ramos E (2019). Use of ergogenic supplements by young athletes in a sports specialized school. J Int Soc Phys Rehabil Med.

[16] Al-Johani WM, Al-Dawood KM, Abdel Wahab MM, Yousef HA (2018). Consumption of vitamin and mineral supplements and its correlates among medical students in Eastern Province, Saudi Arabia. J Family Community Med.

[17] Aguilar-Navarro M, Baltazar-Martins G, Brito de Souza D, Muñoz-Guerra J, Del Mar Plata M, Del Coso J (2021). Gender differences in prevalence and patterns of dietary supplement use in elite athletes. Res Q Exerc Sport.

[18] Froiland K, Koszewski W, Hingst J, Kopecky L (2004). Nutritional supplement use among college athletes and their sources of information. Int J Sport Nutr Exerc Metab.

[19] Ziegler PJ, Nelson JA, Jonnalagadda SS (2003). Use of dietary supplements by elite figure skaters. Int J Sport Nutr Exerc Metab.

[20] Lacerda FM, Carvalho WR, Hortegal EV, Cabral NA, Veloso HJ (2015). Factors associated with dietary supplement use by people who exercise at gyms. Rev Saude Publica.

[21] Svantorp-Tveiten KM, Friborg O, Torstveit MK (2021). Protein, creatine, and dieting supplements among adolescents: use and associations with eating disorder risk factors, exercise-, and sports participation, and immigrant status. Front Sports Act Living.

[22] Dorsch KD, Bell A (2005). Dietary supplement use in adolescents. Curr Opin Pediatr.

[23] Alves C, Lima RV (2009). Dietary supplement use by adolescents. J Pediatr (Rio J).

[24] Kim SH, Keen CL (1999). Patterns of vitamin/mineral supplement usage by adolescents attending athletic high schools in Korea. Int J Sport Nutr.

[25] Stierman B, Mishra S, Gahche JJ, Potischman N, Hales CM (2020). Dietary supplement use in children and adolescents aged ≤19 years - United States, 2017-2018. MMWR Morb Mortal Wkly Rep.

[26] Whitehouse G, Lawlis T (2017). Protein supplements and adolescent athletes: a pilot study investigating the risk knowledge, motivations and prevalence of use. Nutr Diet.

[27] Daher J, El Khoury D, Dwyer JJ (2021). Education interventions to improve knowledge, beliefs, intentions and practices with respect to dietary supplements and doping substances: a narrative review. Nutrients.

[28] Elias S, Saad H, Taib M (2018). Effects of sports nutrition education intervention on sports nutrition knowledge, attitude and practice, and dietary intake of Malaysian team sports athletes. Mal J Nutr.

[29] AlRuthia Y, Balkhi B, Alrasheed M, Altuwaijri A, Alarifi M, Alzahrani H, Mansy W (2018). Use of dietary and performance-enhancing supplements among male fitness center members in Riyadh: a cross-sectional study. PLoS One.

[30] Pomeranz JL, Barbosa G, Killian C, Austin SB (2015). The dangerous mix of adolescents and dietary supplements for weight loss and muscle building: legal strategies for state action. J Public Health Manag Pract.

[31] Ganson KT, Rodgers RF (2022). Problematic muscularity-oriented behaviors: overview, key gaps, and ideas for future research. Body Image.

[32] Crawford C, Boyd C, Brown L (2021). Prioritized research recommendations and potential solutions: addressing gaps surrounding dietary supplement ingredients for boosting brain health and optimizing cognitive performance. Nutr Res.

[33] Walrand S (2018). Dietary supplement intake among the elderly: hazards and benefits. Curr Opin Clin Nutr Metab Care.

[34] Hoffman JR, Faigenbaum AD, Ratamess NA, Ross R, Kang J, Tenenbaum G (2008). Nutritional supplementation and anabolic steroid use in adolescents. Med Sci Sports Exerc.

